# Application of Ophthalmic Electrophysiology in Inflammatory Disorders of Retina and Optic Nerve

**DOI:** 10.3390/jcm13133829

**Published:** 2024-06-29

**Authors:** Minzhong Yu, Shree K. Kurup

**Affiliations:** 1 Department of Ophthalmology, University Hospitals, Case Western Reserve University School of Medicine, Cleveland, OH 44106, USA; 2 Department of Ophthalmic Research, Cleveland Clinic, Cleveland, OH 44195, USA; 3 Department of Ophthalmology, Cleveland Clinic Lerner College of Medicine of Case Western Reserve University School of Medicine, Cleveland, OH 44195, USA

**Keywords:** electroretinogram, electro-oculogram, visual evoked potential, acute posterior multifocal placoid pigment epitheliopathy, acute zonal occult outer retinopathy, Adamantiades–Behçet disease, autoimmune neuro-retinopathy, autoimmune retinopathy birdshot chorioretinopathy, multiple evanescent white dot syndrome, Vogt–Koyanagi–Harada disease

## Abstract

This review covers the utility of electrophysiological studies relevant to inflammatory diseases of the retina in conditions such as acute posterior multifocal placoid pigment epitheliopathy, acute zonal occult outer retinopathy, Adamantiades–Behçet disease, autoimmune retinopathy and neuro-retinopathy, birdshot chorioretinopathy, multiple evanescent white dot syndrome, and Vogt–Koyanagi–Harada disease. Electrophysiological studies can help with the diagnosis, prognostication, evaluation of treatment effects, and follow-up for these conditions.

## 1. Introduction

Inflammatory disorders affecting the retina and optic nerve encompass a diverse group of conditions characterized by inflammation within the ocular structures, leading to visual impairment and potential vision loss. Ophthalmic electrophysiology plays a crucial role in the diagnosis, monitoring, and management of these disorders. This paper reviews the application of ophthalmic electrophysiology in inflammatory conditions of the retina and optic nerve, highlighting its clinical utility and implications for patient care.

Full-field electroretinography (ffERG) is a diagnostic test used to evaluate the function of the retina, the light-sensitive tissue at the back of the eye [1]. It provides valuable information about the overall health and function of the retina, as well as the activity of different types of retinal cells, including photoreceptors and inner retinal cells. ffERG involves the recording of electrical responses generated by the retina in response to flashes of light presented to the eye. During the ffERG test, a series of flashes of light are presented to the eye. These flashes may vary in intensity, duration, and color, allowing the assessment of different aspects of retinal function. The electrical responses generated by the retina are recorded by the electrodes and transmitted to a computer for analysis. The resulting waveform represents the summed activity of the retinal cells in response to the light stimulus. The main components of ffERG includes the a-wave, b-wave, and oscillatory potentials (OPs). The a-wave, the initial negative deflection of the ERG waveform, reflects the hyperpolarization of photoreceptor cells in response to light stimulation. The b-wave is a positive deflection following the a-wave, which primarily reflects the activity of bipolar cells. OPs provide information about the reciprocal synapses between RBC and AII/A17 in the inner retina [2]. The ffERG are tested under dark-adapted and light-adapted conditions, which reflect the functions in the rod and cone pathways, respectively.

Multifocal electroretinography (mfERG) is a specialized diagnostic test used to assess the function of different regions of the retina. Unlike full-field ERG, which provides a global assessment of retinal function, mfERG allows for the localized evaluation of retinal responses to light stimuli across multiple areas of the retina simultaneously. This enables clinicians to detect and localize abnormalities in retinal function with a greater precision. During the mfERG test, the patient is positioned in front of a screen displaying a pattern of light stimuli. These stimuli are presented simultaneously to 61 or 103 discrete areas of the retina, typically arranged in a hexagonal grid pattern. The resulting mfERG waveform consists of a grid of responses, with each response corresponding to a specific region of the retina. Each of the response includes an initial negative deflection (N1), followed by a positive peak (P1) and a subsequent negative peak (P2). The amplitude and implicit time of these components provide information about the function of photoreceptors and inner retinal cells in each region. In addition, the responses from those locations may also be averaged across four quadrants and some concentric rings around the foveola [3,4].

The primary advantage of mfERG is its ability to localize retinal abnormalities to specific regions of the retina. It is valuable in identifying cone-mediated responses in areas of residual photoreceptor function and assessing function loss in both macular and peripheral retinal regions [5]. In addition, it can be used to monitor the changes in retinal function over time, allowing for the early detection of disease progression, as well as for the assessment of the efficacy of treatments for retinal diseases and to monitor the effects of therapeutic interventions on retinal function.

Visual evoked potentials (VEPs) are electrical signals recorded from the brain in response to visual stimuli [6]. They are an essential tool in neuroscience and clinical neurology for assessing the functional integrity of the visual pathway, from the retina to the visual cortex. VEPs provide valuable information about the speed and integrity of visual processing, making them useful in diagnosing and monitoring various visual disorders and neurological conditions. VEPs are generated by the sequential activation of different parts of the visual pathway in response to visual stimuli. When light enters the eye, it is projected onto the retina, where photoreceptor cells convert it into neural signals. These signals are transmitted via the optic nerve to the visual cortex at the back of the brain, where visual processing occurs. The visual cortex then generates electrical activity in response to the incoming visual stimuli. During the VEP test, electrodes are placed on the scalp at specific locations corresponding to different regions of the visual cortex. The electrical signals generated by the brain in response to the visual stimuli are amplified, filtered, and recorded by the electrophysiologic system. These signals are then analyzed to extract the VEP waveform. The VEP waveform typically consists of several components, each reflecting different stages of visual processing. The Negative Peak 1 (N1) represents the initial neural response to the visual stimulus and is believed to originate primarily from the primary visual cortex (V1). The Positive Peak 1, a positive deflection in the waveform, known as P1, is thought to reflect further processing within the visual cortex, including feature extraction and pattern recognition. The Negative Peak 2 (N2) is another negative deflection in the waveform, occurring after P1. It may reflect additional processing within the visual cortex or feedback from higher-order visual areas. The Positive Peak 2 (P2) is a later positive deflection in the waveform, which may represent a more complex cognitive processing related to attention and perception. The latency and amplitude of these components can provide valuable information about the integrity and efficiency of the visual pathway.

VEPs are used clinically to assess various visual disorders and neurological conditions, including optic neuritis, multiple sclerosis, amblyopia, glaucoma, and cortical blindness. Changes in VEP latency or amplitude can indicate abnormalities in visual processing and help diagnose and monitor these conditions. Moreover, VEPs are also used in research settings to investigate visual perception, attention, and cognition. They provide insights into how the brain processes visual information and can be used to study the effects of aging, development, and neurological disorders on visual function.

Electro-oculography (EOG) is a method used to record the electrical activity of the muscles that control eye movement [7]. This technique relies on the fact that the posterior of the eye has a negative charge relative to the cornea, known as standing potential, which is correlated to the transepithelial potential (TEP) of the retinal pigment epithelium (RPE). TEP changes with dark-adaptation and light-adaptation. A potential difference between the inner canthus and outer canthus is changed during eye movement, which can be used for the indirect measurement of the standing potential with a fixed-angle eye movement. In EOG recordings, the light-peak refers to the peak amplitude of the EOG signal following exposure to a light stimulus, while the dark-trough corresponds to the lowest point in the signal during periods of darkness. The light-peak reflects the hyperpolarization of the RPE cells in response to light stimulation, whereas the dark-trough represents the baseline activity of the RPE in the absence of light. The EOG light-peak to dark-trough ratio is calculated by dividing the peak amplitude of the EOG signal during light stimulation by the trough amplitude during darkness. A higher ratio indicates a more robust RPE response to light and a healthier retinal function, as the amplitude of the response to light should ideally exceed the baseline activity during darkness. This ratio has clinical relevance in the assessment of various retinal disorders, particularly those affecting the RPE. Changes in the EOG light-peak to dark-trough ratio can provide early indications of RPE dysfunction, allowing for the timely intervention and monitoring of disease progression. Furthermore, the EOG light-peak to dark-trough ratio can be used to evaluate the efficacy of therapeutic interventions targeting RPE function, such as pharmacological agents or gene therapies. By tracking changes in this ratio over time, clinicians can assess the treatment response and adjust the therapeutic strategies accordingly.

This paper summarizes the distinctive features and findings of multiple evanescent white dot syndrome, acute posterior multifocal placoid pigment epitheliopathy, birdshot chorioretinopathy, autoimmune retinopathy, and neuro-retinopathy. It initially discusses the genetic approach and clinical presentation of each condition before exploring the role of electrophysiological testing in their diagnosis and evaluation.

## 2. Clinical Applications of Electrophysiology

### 2.1. Acute Posterior Multifocal Placoid Pigment Epitheliopathy (APMPPE)

Acute posterior multifocal placoid pigment epitheliopathy (APMPPE), an inflammatory chorioretinopathy initially identified by Gass in 1968 [8], is a rare inflammatory chorioretinopathy characterized by multiple yellow-white placoid subretinal lesions predominantly located in the posterior pole. Symptoms manifest swiftly with the emergence of central or paracentral scotomas, accompanied by a sudden onset of blurred vision. Visual acuity typically deteriorates to 20/40 or even to the extent of finger counting. Preceding vision impairment, patients may experience photopsia. While commonly affecting both eyes, symptoms might debut unilaterally, separated by a few days. An examination of the fundus reveals bilateral yellow-white placoid lesions varying in size from one- to two-disc diameters at the choroid and RPE levels. Within three weeks of the onset, new lesions may arise in the periphery [9,10,11].

APMPPE often resolves spontaneously within 4 to 8 weeks, with some cases extending up to 6 months. Most patients eventually recover visual acuity of 20/40 or better, though the prognosis worsens with foveal involvement [12]. Prior to APMPPE development, nearly one-third of patients report a preceding viral or flu-like illness [13]. Complications such as retinal vasculitis, optic disc edema, subhyaloid hemorrhage, choroidal neovascular membrane formation, and vein occlusion have all been associated with this condition [11,14].

The etiology of APMPPE remains elusive, although speculation suggests its emergence as a consequence of delayed-type hypersensitivity vasculitis. Inflammation at the choriocapillaris level leads to hypoperfusion and ischemia in the RPE, subsequently resulting in the loss of photoreceptors and hyperpigmentation in later stages [15,16]. Genetic predisposition linked to HLA-B7 and HLA-DR2 haplotypes may influence APMPPE susceptibility. Additionally, viral infection has been proposed as another potential association [17,18].

While the clinical presentation and management of APMPPE have been extensively studied, the role of electrophysiology in understanding its pathophysiology and guiding treatment remains a topic of growing interest.

ffERG is a valuable tool for assessing retinal function in APMPPE. During the acute phase, ERG findings typically show marginal reductions in both the a- and b-wave amplitudes, reflecting the dysfunction of photoreceptors and bipolar cells [19]. In addition, it is observed that the ERG response of S-cones were reduced more than the mixed L- and M- cones in the affected eyes, and the ratio of the S-cone response amplitude of the affected eyes to that of the normal fellow eyes is significantly lower than the ratio for the L- and M-cone [20]. However, as the disease progresses to the scar stage, ERG may demonstrate a recovery of amplitude, indicating the partial restoration of retinal function. These findings suggest that, while the initial insult in APMPPE affects retinal function, there is potential for functional recovery over time.

mfERG provides the spatially resolved assessment of retinal function and has been utilized to evaluate APMPPE. During the acute phase, mfERG may reveal a notable decline in the response density of the central retina, corresponding to the extent of retinal involvement by the placoid lesions. However, similar to ERG, mfERG findings may improve in the scar stage, indicating the partial recovery of retinal function. The ability of mfERG to localize and quantify retinal dysfunction makes it a valuable tool for monitoring disease progression and treatment response in APMPPE [21].

### 2.2. Acute Zonal Occult Outer Retinopathy (AZOOR)

Acute zonal occult outer retinopathy (AZOOR) is a rare and enigmatic retinal disorder characterized by sudden vision loss, scotomas, and electrophysiological abnormalities. Despite its rarity, AZOOR poses significant diagnostic and management challenges due to its varied clinical presentation and poorly understood pathophysiology. Unlike genetic conditions such as retinitis pigmentosa, AZOOR does not have a hereditary component. Instead, it displays distinctive alterations in the fundus and imaging characteristics that signify dysfunction in the outer retina [22]. Gass was the pioneer in delineating the characteristics of acute zonal occult outer retinopathy (AZOOR) in 1993 [23]. This condition is defined as an acquired inflammatory disorder marked by symptoms such as photopsia, central scotomas, and the rapid loss of outer retinal function, characterized by the distinct demarcation between affected and unaffected retinal regions. Several authors have suggested that AZOOR, along with other conditions such as multiple evanescent white dot syndrome (MEWDS), ocular histoplasmosis syndrome (OHS), multifocal choroiditis, acute macular neuroretinopathy (AMN), acute idiopathic blind spot enlargement syndrome (AIBSES), punctate inner choroiditis (PIC), and autoimmune retinopathy (AIR), may represent distinct manifestations of a shared disease spectrum, because they have a large clinical and symptomatic overlap [24,25,26,27,28]. The autoimmune basis is supported by the presence of anti-retinal and anti-RPE autoantibodies in AZOOR patients [29,30,31].

Recent advancements in AZOOR imaging, particularly through fundus autofluorescence (FAF), have provided significant insights. Even in cases where traditional ophthalmoscopic signs are absent or minimal, anomalous FAF patterns are consistently observed in AZOOR patients, particularly around the optic nerve head and along vascular arcades [32,33,34,35,36].

Electrophysiological tests have emerged as indispensable tools in unraveling the mysteries surrounding AZOOR and provide valuable insights into the functional integrity of the retina, aiding in the identification of characteristic abnormalities associated with AZOOR.

In AZOOR, ffERG findings often reveal characteristic abnormalities, including attenuated or absent photopic and scotopic responses, indicating the dysfunction of both the cone and rod photoreceptors. The amplitude reduction is typically more severe than the implicit time prolongation, indicating a primary photoreceptor dysfunction rather than a post-receptoral defect [37,38]. Additionally, evidence of spontaneous recovery or disease stabilization may be observed in some patients during follow-up assessments, providing valuable prognostic information.

Additionally, mfERG offers the localized assessment of retinal function, aiding in the identification of zonal areas of dysfunction in AZOOR. The presence of localized amplitude reductions or delays in mfERG responses corresponds to the distribution of visual field defects, facilitating the precise localization of retinal pathology [38].

Electrophysiology not only aids in the diagnosis of AZOOR but also guides therapeutic strategies and disease monitoring. The detection of localized retinal dysfunction using mfERG facilitates targeted treatment approaches such as focal laser photocoagulation or immunomodulatory therapy aimed at preserving functional retinal tissue and preventing disease progression. The longitudinal assessment of electrophysiological parameters enables the monitoring of treatment response and disease evolution over time. Serial ERG and mfERG evaluations provide objective measures of treatment efficacy and disease activity, guiding therapeutic adjustments as needed.

### 2.3. Adamantiades–Behçet Disease

Adamantiades–Behçet disease, commonly referred to as Behçet’s disease (BD), is a complex multisystemic inflammatory disorder characterized by recurrent oral and genital ulcers, ocular inflammation (uveitis), skin lesions, and the involvement of various organs such as the gastrointestinal tract, central nervous system, and vascular system. The exact etiology and pathogenesis of Behçet’s disease remain incompletely understood, but both genetic predisposition and dysregulated immune responses are believed to play significant roles.

Genetic factors are thought to contribute to the susceptibility to BD, with certain human leukocyte antigen (HLA) alleles being strongly associated with increased risk. HLA-B51, in particular, has been identified as a major genetic susceptibility factor, with individuals carrying this allele being significantly more likely to develop Behçet’s disease. However, the presence of HLA-B51 alone is not sufficient to cause the disease, suggesting that additional genetic and environmental factors are involved in its pathogenesis. While HLA-B51 is not particularly helpful for diagnosing the condition, it plays a significant role in identifying clinical phenotypes within this diverse disease.

Immune dysregulation is central to the pathogenesis of Behçet’s disease, with the aberrant activation of both innate and adaptive immune responses contributing to tissue inflammation and damage. Dysregulated T-cell responses, including Th1, Th17, and CD8+ T-cell activation, have been implicated in the pathogenesis of BD. Th1 cells secrete pro-inflammatory cytokines such as interferon-gamma (IFN-γ) and tumor necrosis factor-alpha (TNF-α), which promote inflammation and activate immune cells. Th17 cells produce interleukin-17 (IL-17), a key cytokine involved in the recruitment of neutrophils and the amplification of inflammation. CD8+ T cells, also known as cytotoxic T lymphocytes, contribute to tissue damage by directly attacking and killing target cells.

In addition to T-cell-mediated immune responses, the dysregulation of the innate immune system, including the activation of the inflammasome pathway, has been implicated in Behçet’s disease pathogenesis. The inflammasome is a multiprotein complex that plays a critical role in the activation of inflammatory responses by processing and releasing pro-inflammatory cytokines, such as interleukin-1β (IL-1β) and interleukin-18 (IL-18). The aberrant activation of the inflammasome pathway has been observed in BD, leading to the increased production of pro-inflammatory cytokines and perpetuation of inflammation.

Furthermore, vascular involvement is a prominent feature of BD, with endothelial dysfunction and vasculitis contributing to the development of systemic manifestations such as thrombosis and aneurysms. Endothelial injury and the activation of the coagulation cascade may result from the direct effects of inflammatory mediators, immune complex deposition, or the direct invasion of vascular tissues by immune cells.

Visual electrophysiology serves as a valuable tool in the assessment of ocular involvement in BD, aiding in the diagnosis, monitoring, and management of ocular manifestations. Given that ocular inflammation is a hallmark feature of BD and can lead to significant visual impairment if left untreated, visual electrophysiological tests provide objective measures of retinal function and integrity, complementing clinical examination findings.

In BD, ffERG may demonstrate abnormalities reflective of retinal dysfunction, such as reductions in amplitude and the prolongation of the implicit time. These ERG abnormalities can occur due to direct retinal inflammation, the involvement of the retinal pigment epithelium (RPE), or damage to photoreceptor cells. ERG findings in BD correlate with the severity and activity of ocular inflammation, providing valuable insights into disease progression and guiding treatment decisions [39].

mfERG reveals focal or diffuse abnormalities corresponding to areas of active inflammation or damage within the retina in BD, aiding in the early detection and management of ocular complications. In the study of Stübiger et al., the mfERG amplitude was reduced and the implicit time was prolonged in the central retina with macular edema, which was significantly improved 8 months after IFN α2a therapy, except in advanced stages [40].

### 2.4. Autoimmune Retinopathy and Neuro-Retinopathy

Autoimmune retinopathy (AIR) and neuro-retinopathy (AINR, also known as autoimmune-related retinopathy and optic neuropathy, or ARRON) represent a spectrum of acquired retinal and/or optic nerve disorders driven by autoantibody (AAb)-mediated inflammation and tissue damage [28,29,41,42,43,44,45,46,47,48,49,50,51,52,53,54,55]. Classified into paraneoplastic (pAIR/AINR) and non-paraneoplastic (npAIR/AINR) forms, npAIR cases predominate, with only approximately 20–25% exhibiting a paraneoplastic association [28,56,57]. The paraneoplastic forms encompass cancer-associated retinopathy (CAR) and melanoma-associated retinopathy (MAR), with emerging entities like the basal-cell-carcinoma-associated retinopathy and optic neuropathy (BARN) complex [58] and teratoma-associated CAR [59,60]. The onset age of AIR varies, predominantly affecting adults, while childhood onset is documented [61]. The AIR symptoms can progress rapidly which is asymmetric between the two eyes (especially in npAIR), or can also progress slowly and chronically, mimicking retinal dystrophies, necessitating diagnostic testing [28,52,56,57].

**Figure 1:**
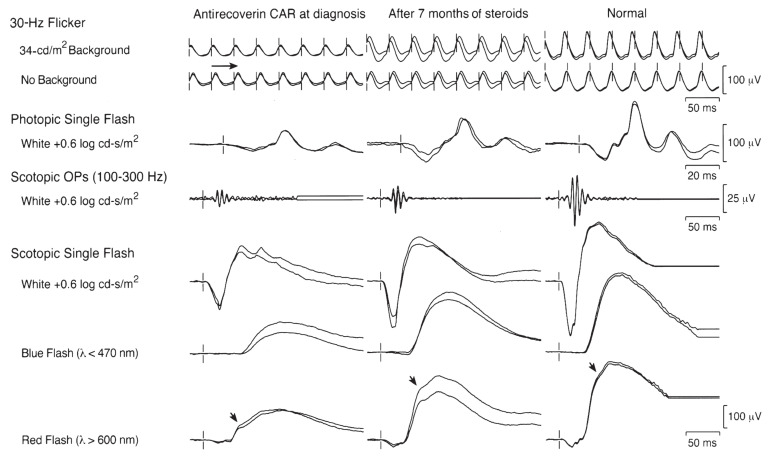
ffERG with expanded International Society for Clinical Electrophysiology of Vision protocol of a patient with antirecoverin CAR at the time of autoimmune retinopathy diagnosis and 7 months after corticosteroid therapy. The responses from the right and left eyes are superimposed. Cone and rod ERG responses in the patient are mildly reduced compared to the normal subject, and the amplitudes of OPs are also severely reduced. After corticosteroid treatment for 7 months, ffERG shows a significant increase in amplitudes of rod responses, scotopic cone responses to red flash (arrows), photopic cone responses to single flash and 30 Hz flicker, and, to a lesser extent, scotopic bright white flash and scotopic OPs. The rod and cone implicit times, including 30 Hz flicker (horizontal arrow designates peak timing), remained mildly prolonged [42].

In pAIR, visual symptoms may precede cancer detection. Ophthalmologists, particularly visual electrophysiologists, play a pivotal role in recognizing pCAR, potentially uncovering occult cancers and saving lives. While antiretinal autoantibodies are suggestive, ancillary tests including electrophysiology and imaging are essential for the diagnosis and management of AIR and AINR [43,50,62,63]. These conditions, characterized by the autoimmune-mediated damage to retinal structures and pathways, present unique challenges in both understanding their pathophysiology and devising effective treatment strategies. In both pAIR and npAIR, ffERG shows a functional decrease in the rod and cone photoreceptor, as well as in bipolar cells, which can be recovered notably after treatment (Figure 1). mfERG was used to show the functional change in the retina in the tested field in some of those patients, which presented different types of abnormalities [42,57]. An electronegative mixed response is common in MAR patients, due to the presence of AAbs directed against bipolar cell antigens [64,65,66]. The EOG light peak-to-dark trough ratio is reduced in the patients with anti-bestrophin autoantibodies [67].

Those displaying characteristics indicative of optic nerve or pathway involvement, either as the primary cause of autoimmune-mediated vision loss or as an additional factor in cases of AIR, often exhibit positive serologies for anti-optic nerve antibodies and/or anti-retinal antibodies. Those antibodies, such as antibodies to recoverin, anti-enolase-alpha autoantibodies, and TRPM1 autoantibodies, may show a distinct staining pattern with different specificity and sensitivity on diagnostic retinal immunohistochemistry, particularly affecting the RGCL [51]. These abnormalities, observable under microscopic examination, can suggest conditions like juxtapapillary chorioretinitis. Therefore, the evaluation of the retinal ganglion cell layer (RGCL), retinal nerve fiber layer (RNFL), and optic nerve function, utilizing both direct and indirect measures, should be performed for assessing patients with AIR. It was observed that, in AINR, the pattern VEP P100 implicit time was prolonged, and the amplitude was reduced in AINR [68].

Ophthalmic electrophysiology represents a valuable adjunctive tool in the diagnosis, monitoring, and management of AIR and AINR. By providing insights into the functional integrity of retinal structures and pathways, electrophysiological techniques aid in understanding the pathophysiology of these complex conditions and guide clinical decision-making for optimal patient care. Further research exploring the utility of emerging electrophysiological modalities holds promise for advancing our understanding and treatment of these sight-threatening diseases.

### 2.5. Birdshot Chorioretinopathy

Birdshot chorioretinopathy (BSCR) is a rare uveitis, characterized by acute-onset, recurring, chronic, bilateral, diffuse, orange- to cream-colored choroidal lesions with minimal vitritis, typically located in the posterior pole and the mid-periphery of the retina. Its name derives from the distinctive fundus lesions resembling the scattered pattern of birdshot pellets. Patients often present with blurred vision, floaters, nyctalopia, dyschromatopsia, glare, and photopsia [69]. Comprising 1–2% of uveitis cases, BSCR predominantly affects middle-aged Caucasian females, aged between 40 and 60 years old, and exhibits a strong association with the HLA-A29-positive in 80–98% of patients compared to 7% in the general population. Despite this genetic link, the precise etiology remains elusive, with some hypotheses implicating an autoimmune reaction targeting arrestin as a potential trigger [70]. Histopathologically, it is associated with the lymphocytic infiltration of the choroid and retina, leading to progressive retinal dysfunction and atrophy. The characteristic lesions resembling the dispersion pattern of birdshot pellets give rise to the condition’s moniker. These chorioretinal lesions, ranging from 0.25- to 0.50-disc diameters, tend to cluster around the optic nerve and posterior pole, radiating outward, with a predilection for the inferonasal peripapillary region. The distribution of lesions may vary widely, encompassing the entire fundus or sparing the macula entirely, often exhibiting asymmetry between the two eyes [69,70,71,72].

Early-stage BSCR manifests with retinal vascular leakage, often observed at or away from the optic disc, and hypofluorescent lesions on indocyanine green (ICG) angiography, providing valuable diagnostic clues. In subsequent stages, characterized as the “middle stage”, prominent birdshot lesions become evident on fluorescein angiography and fundus autofluorescence (FAF). The progression to advanced stages may feature complications such as cystoid macular edema, disc leakage, retinal pigment epithelium (RPE) alterations, retinal atrophy, vascular attenuation, optic nerve atrophy, and subretinal neovascularization, significantly impacting visual acuity.

**Figure 2:**
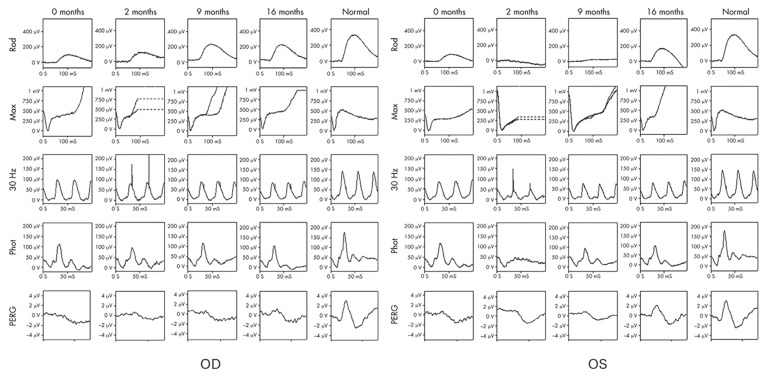
ffERGs of a 55-year-old HLA-A29-positive woman with BSCR before and after steroid therapy. Before the treatment, b-wave, 30 Hz flicker responses, and PERG were reduced, which were more significant in scotopic ERG. Electronegative ERGs showed reduction in inner retinal dysfunction that occurs after phototransduction. After treatment for 16 months, those reductions were significantly recovered [73].

Electrophysiological testing, particularly ERG, can aid in the diagnosis of BSCR, especially in cases where clinical findings are inconclusive or atypical. The characteristic ERG pattern of generalized retinal dysfunction helps distinguish BSCR from other uveitic entities, guiding the appropriate management strategies. The ffERG of BSCR often demonstrates a reduction in both the rod and cone responses, reflecting generalized retinal dysfunction. However, the extent of involvement may vary among patients, ranging from subclinical abnormalities to severe dysfunction. Notably, abnormalities in ffERG, particularly an electronegative mixed response [71], are common even in the absence of characteristic lesions. Aberrations in electro-oculography (EOG) parameters, including a reduced light peak and light peak-to-dark trough ratio, suggest the inflammatory involvement of the RPE at the chorioretinal interface [71]. Furthermore, serial electrophysiological assessments allow for the longitudinal monitoring of retinal and optic nerve function in BSCR patients. Changes in ERG responses over time can provide valuable insights into disease progression and treatment effect (Figure 2), facilitating a timely intervention to prevent irreversible visual loss.

Unlike self-resolving conditions, birdshot chorioretinopathy demands intervention to prevent progressive vision loss. The initial treatment typically involves oral or intravitreal/subtenon steroids, followed by systemic immunomodulatory therapy (IMT) in most cases. IMT has demonstrated efficacy in stabilizing or improving visual acuity and visual field deficits in up to 89% of patients [69,74,75]. Recent advancements in biologic IMT options have shown promise in refractory cases, emphasizing the importance of early detection and diagnosis in optimizing treatment outcomes. Electrophysiologic tests serve as an objective tool for evaluating treatment effects in BSCR.

### 2.6. Multiple Evanescent White Dot Syndrome

Multiple evanescent white dot syndrome (MEWDS) is a rare and self-limiting inflammatory chorioretinopathy. First documented in 1984 by Jampol et al. [76], MEWDS is characterized by transient, multifocal, white-yellow lesions at the level of the outer retina and retinal pigment epithelium (RPE). It presents with sudden, painless unilateral vision loss, often dropping to 20/200, accompanied by visual field defects, retinal pigment epithelium (RPE) staining, and fluorescein leakage from disc capillaries. Additionally, indocyanine green angiography typically reveals peripapillary hypofluorescence. The symptoms include photopsias, floaters, and dyschromatopsia [77,78,79,80,81]. An ocular fundus examination reveals numerous white dots, ranging from 100 to 200 μm in width at the outer retina or RPE, often observed subclinically and best visualized through fundus autofluorescence (FAF) imaging. Optical coherence tomography (OCT) demonstrates the disruption of outer retinal layers in affected areas [82]. Predominantly affecting females aged 15 to 50, MEWDS may present with flu-like prodromes in one-third to half of the cases. Typically self-limiting, the syndrome resolves spontaneously within three to nine weeks, with most patients experiencing visual acuity recovery. Scotomata and photopsias diminish over time, while fundus lesions may progress to chorioretinal scarring or moderate pigment mottling. Recurrence occurs in around 10% of cases, with persistent blind spot enlargement noted in some patients [77,78,79,80,81]. While the clinical features of MEWDS have been well-described, the role of electrophysiology in elucidating its pathophysiology and guiding management remains an area of interest.

ERG serves as a valuable tool in assessing retinal function in MEWDS. During the acute phase, ERG findings often demonstrate reduced a- and b-wave amplitudes in rod and cone ERGs, indicating the dysfunction of both photoreceptors and bipolar cells. Additionally, a delay in implicit times may be observed, reflecting impaired retinal processing. As the disease progresses, ERG abnormalities tend to resolve, correlating with clinical improvement. These findings suggest that the primary insult in MEWDS affects retinal function, with the potential for recovery over time [83].

mfERG provides a spatially resolved assessment of retinal function and has been utilized to evaluate MEWDS. During the acute phase, mfERG may reveal reduced response densities in the affected areas, corresponding to the location of the white dot lesions. However, as the disease resolves, mfERG responses typically return to baseline, indicating the restoration of retinal function. The ability of mfERG to localize and quantify retinal dysfunction makes it a valuable tool for monitoring disease progression and treatment response in MEWDS [83,84,85].

EOG evaluates the functional integrity of the RPE and has been investigated in MEWDS. During the acute phase, EOG responses may demonstrate abnormalities, reflecting the dysfunction of the RPE. However, as the disease resolves, the EOG recordings tend to normalize, suggesting the recovery of RPE function. Given the role of the RPE in maintaining retinal homeostasis, EOG provides insights into the pathophysiology of MEWDS and may aid in predicting visual outcomes.

The application of electrophysiological techniques, including ERG, mfERG, and EOG, offers valuable insights into the pathophysiology and progression of MEWDS. These techniques provide objective measures of retinal and RPE function, aiding in the diagnosis, monitoring, and management of MEWDS. Furthermore, the ability of electrophysiology to assess the treatment response and predict visual outcomes underscores its importance in the comprehensive evaluation of MEWDS patients.

### 2.7. Vogt–Koyanagi–Harada Disease

Vogt–Koyanagi–Harada (VKH) disease is a multisystemic autoimmune disorder characterized by the inflammation of melanocyte-rich tissues, particularly the eyes, ears, skin, and meninges. The exact etiology of VKH disease remains elusive, but it is believed to involve a complex interplay of genetic predisposition, environmental triggers, and aberrant immune responses targeting melanocytes. One prevailing theory suggests that VKH disease arises from an autoimmune reaction against melanocytes, which are pigment-producing cells found in various tissues throughout the body. In genetically susceptible individuals, the exposure to certain environmental triggers, such as viral infections or environmental antigens resembling melanocyte proteins, may lead to the activation of autoreactive T lymphocytes. These activated T cells then infiltrate melanocyte-rich tissues, triggering an inflammatory cascade and causing tissue damage.

The central to the pathogenesis of VKH disease is the breakdown of immune tolerance to melanocyte antigens. Studies have identified several candidate antigens expressed by melanocytes that may serve as targets for autoreactive T cells in VKH disease, including tyrosinase-related protein 1 (TRP-1) and TRP-2. The recognition of these melanocyte antigens by autoreactive T cells leads to the activation of cytotoxic T lymphocytes and the release of pro-inflammatory cytokines, such as interleukin-17 (IL-17) and interferon-gamma (IFN-γ), which perpetuate inflammation and recruit additional immune cells to the site of injury. In addition to T-cell-mediated mechanisms, humoral immune responses may also contribute to the pathogenesis of VKH disease. Autoantibodies targeting melanocyte antigens or components of the blood–retinal barrier have been detected in the serum and ocular fluids of patients with VKH disease. These autoantibodies may exacerbate tissue damage by promoting complement activation, immune complex deposition, and the recruitment of inflammatory cells. Furthermore, genetic factors play a significant role in VKH disease susceptibility, with certain human leukocyte antigen (HLA) alleles, such as HLA-DR4 and HLA-DRB1*0405, being strongly associated with increased risk. These HLA alleles are thought to influence the presentation of melanocyte antigens to T cells and modulate immune responses, contributing to the development and progression of VKH disease.

Diagnosing Vogt–Koyanagi–Harada (VKH) disease can be challenging due to its heterogeneous clinical presentation and the absence of specific diagnostic tests. However, a combination of clinical findings, ancillary tests, and the exclusion of other conditions can aid in establishing the diagnosis. Clinical manifestations of VKH disease typically involve various systems, including the eyes, ears, skin, and meninges. Ocular involvement is often prominent and may include bilateral granulomatous uveitis, serous retinal detachments, optic disc swelling (papillitis), and diffuse choroiditis. Patients may present with blurred vision, photophobia, eye pain, or visual field deficits. Ocular symptoms are often accompanied by auditory disturbances (such as tinnitus or hearing loss), skin changes (such as alopecia or vitiligo), and neurological symptoms (such as headache or meningismus), reflecting the multisystemic nature of the disease.

Ancillary tests are valuable adjuncts in the diagnosis of VKH disease and may help confirm the clinical suspicion. Optical coherence tomography (OCT) can detect characteristic findings such as subretinal fluid, choroidal thickening, and the disruption of the retinal pigment epithelium (RPE), aiding in the assessment of disease activity and response to treatment. Fluorescein angiography (FA) and indocyanine green angiography (ICGA) can reveal signs of choroidal inflammation, such as multiple pinpoint leaks (pooling) or early choroidal hyperfluorescence, which are consistent with VKH disease. Laboratory investigations may also be useful in supporting the diagnosis of VKH disease and ruling out other conditions. Serological tests for autoimmune markers, such as antinuclear antibodies (ANA) or rheumatoid factor (RF), may be performed to assess for underlying autoimmune diseases that can mimic VKH disease. Additionally, a cerebrospinal fluid (CSF) analysis may reveal lymphocytic pleocytosis and elevated protein levels, indicative of meningeal inflammation, a hallmark feature of VKH disease. Importantly, the diagnosis of VKH disease requires the exclusion of other conditions that can present with similar clinical features, such as sarcoidosis, syphilis, tuberculosis, and other infectious or inflammatory uveitic syndromes. A comprehensive evaluation, including a detailed medical history, thorough physical examination, and appropriate diagnostic testing, is essential in order to differentiate VKH disease from its mimickers and initiate timely and appropriate management.

In the patients with VKH disease, electrophysiological assessments play a crucial role in understanding the pathophysiology and monitoring the progression of VKH disease, particularly in the ocular manifestations.

**Figure 3:**
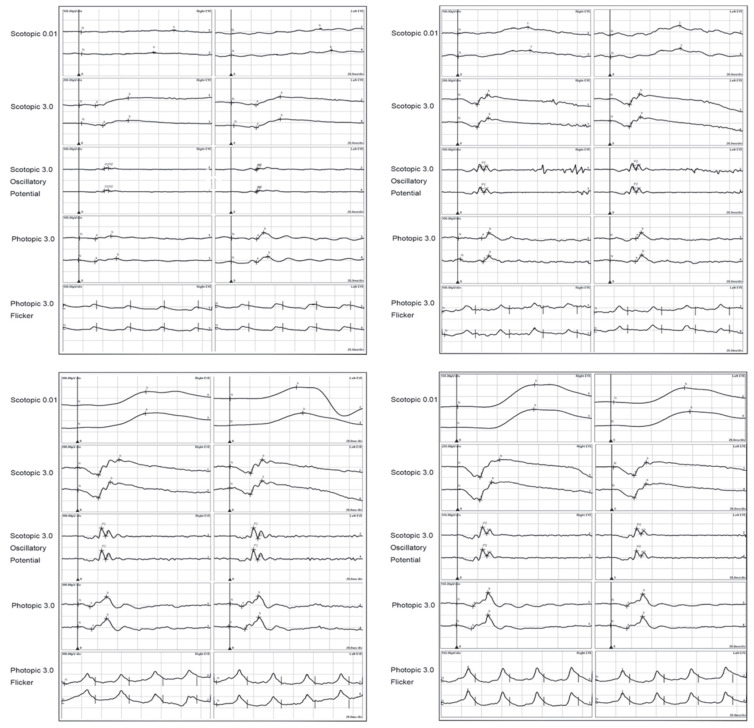
ffERGs of a 38-year-old male with VKH during treatment. (**Top left**): before treatment; (**top right**): 2 months after treatment; (**bottom left**): 6 months after treatment; and (**bottom right**): normal control. Both scotopic and photopic ERG responses were recovered gradually with treatment [88].

ffERG is a key electrophysiological tool used in the evaluation of VKH disease and the treatment effect. In VKH disease, ERG findings typically demonstrate a reduction in both rod and cone responses, reflecting the widespread retinal dysfunction associated with the condition. A significant reduction in rod and cone ERG amplitudes and a mild increase in the a- and b-wave implicit times are correlated with the severity of ocular inflammation and the extent of retinal involvement, and these parameters can be recovered in treatment [86,87,88]. Figure 3 shows the recovery of ffERG responses with corticosteroids combined with other immunosuppressive agents in a VKH patient.

**Figure 4:**
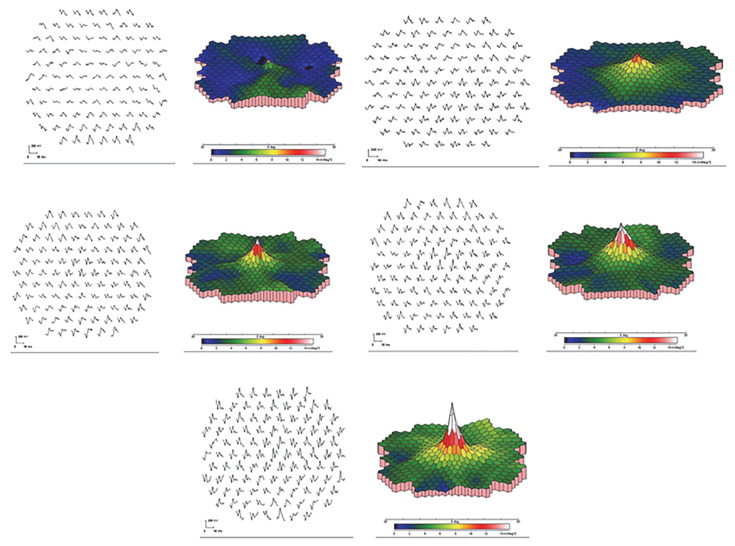
mfERG in the left eye of a 39-year-old male with VKH during treatment. (**Top left**): before treatment; (**top right**): 1 month after treatment; (**middle left**): 3 months after treatment; (**middle right**): 6 months after treatment; and (**bottom**): 12 months after treatment. The response densities gradually increase during treatment, which reflects effective treatment [90].

In VKH disease, mfERG may reveal the localized areas of dysfunction corresponding to the areas of active inflammation or damage within the retina. This spatial mapping of retinal function can aid in the early detection of subclinical disease activity and provide valuable information for treatment monitoring [89]. In addition, mfERG indicates that the response densities recovered gradually during the treatment with corticosteroids combined with other immunosuppressive agents (Figure 4), and there is a delay of the recovery of macular function compared to visual acuity after the treatment with immunosuppressive agents [90].

In summary, electrophysiological assessments, including ERG and mfERG, play an integral role in the evaluation and management of VKH disease. These tests provide valuable information regarding the functional integrity of the retina, aiding in the diagnosis, monitoring of disease activity, and assessment of treatment response. Incorporating electrophysiological findings into the comprehensive management of VKH disease can help optimize patient outcomes and preserve visual function.

## 3. Conclusions

Ophthalmic electrophysiology plays a crucial role in the evaluation and management of inflammatory disorders affecting the retina and optic nerve. It offers an objective and distinct evaluation of retinal function (Table 1) alongside clinical assessments such as visual acuity, color vision, contrast sensitivity, and visual field tests. When combined with imaging modalities like optical coherence tomography (OCT), electrophysiological testing can provide valuable insights for diagnosis, monitoring disease progression, and assessing treatment response. Advances in electrophysiological techniques, including the development of more sensitive protocols and novel biomarkers, hold promise for further enhancing the utility of ophthalmic electrophysiology in inflammatory ocular conditions. Continued research efforts aimed at elucidating the pathophysiology of these disorders and refining electrophysiological testing methods are essential for improving clinical outcomes and optimizing patient care. While electrophysiological testing provides valuable insights into the diagnosis and monitoring of inflammatory diseases of the retina and optic nerve, it has notable limitations. One significant constraint is the potential for overlap in electrophysiological findings among different diseases, particularly during acute stages, which can complicate precise differentiation. Additionally, the accessibility of electrophysiological testing is limited, as not all practices or clinics possess the necessary equipment and expertise to perform these tests on an ad hoc basis. This restricts the widespread applicability and utility of electrophysiological assessments in routine clinical settings. Furthermore, the sensitivity and specificity of these tests can vary, potentially leading to inconclusive or ambiguous results. As multimodal retinal imaging becomes increasingly important, it is essential to consider its integration with electrophysiological testing to enhance diagnostic accuracy and patient management. Highlighting these limitations underscores the need for a comprehensive, multimodal approach in the evaluation of inflammatory retinal and optic nerve diseases.

## Figures and Tables

**Table 1:** Change in electrophysiology in some inflammatory diseases of retina and optic nerve.

Disease	ffERG	mfERG	S-cone ERG	VEP	EOG
APMPPE	Reduction in photopic/scotopic a- and b-waves	Reduction in responses in the affected loci	Reduction		Reduction
AZOOR	Reduction and delay in photopic/scotopic a- and b-waves	Reduction and delay in local responses			
BD	Reduction in photopic/scotopic a- and b-waves	Reduction in responses in the affected loci			
AIR	Reduction and delay in photopic/scotopic a- and b-waves	Reduction in responses, often in central field			Reduction
AINR				Delay and reduction	Reduction
BSCR	Reduction in photopic/scotopic a- and b-waves, more obvious in b-wave reduction				
MEWDS	Reduction in photopic/scotopic a- and b-waves	Reduction in responses in tested field			
VKH	Reduction and delay in photopic/scotopic a- and b-waves	Reduction in responses in the affected loci			

## Data Availability

Not applicable.
